# Reduced fetal cerebral oxygen consumption is associated with abnormal white matter in newborns with congenital heart disease

**DOI:** 10.1186/1532-429X-17-S1-P201

**Published:** 2015-02-03

**Authors:** Prakash Muthusami, Sujana Madathil, Susan Blaser, Edgar Jaeggi, Lars Grosse-Wortmann, Shi-Joon Yoo, John Kingdom, Edward J Hickey, John G Sled, Christopher Macgowan, Steven Miller, Mike Seed

**Affiliations:** The Hospital for Sick Children, Toronto, ON Canada; Mount Sinai Hospital, Toronto, ON Canada

## Background

Congenital heart disease (CHD) is associated with brain dysmaturation, increased risk of perioperative white matter (WM) injury and neurodevelopmental delay (1). Fetal Doppler studies have shown altered cerebrovascular flow dynamics in CHD, suggesting ‘brain-sparing physiology'. We sought to determine whether postnatal cerebral WM microstructural abnormality relates to abnormal fetal hemodynamics.

## Methods

This prospective IRB approved study included 15 fetuses with CHD and 25 normal fetuses at 36 gestational weeks, who underwent phase-contrast MRI using metric optimized gating and T2-mapping for MR oximetry in the major vessels, according to our previously published technique (2). Neonatal MRI was performed at 5 days (SD 5 days) including diffusion tensor imaging and axial T2W-FSE. Apparent diffusion coefficient (ADC) values were measured in the centrum semiovale (CSO), frontal and parietal deep WM, and mean cerebral WM-ADC was calculated. Visual scoring of WM was performed using a 0 - 6 scale (number of T2-hyperintense regions).

## Results

**Figure 1:**
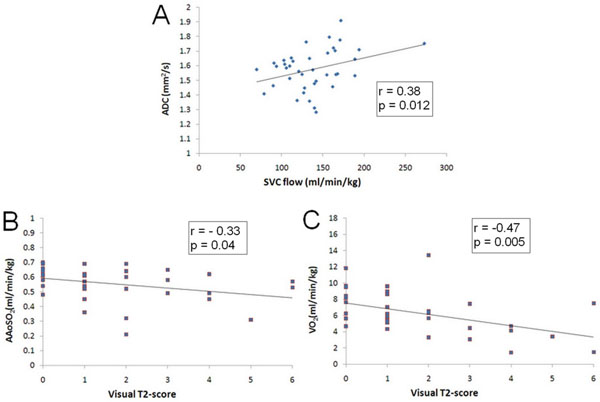
Relations between fetal hemodynamics and neonatal white matter (WM) changes in congenital heart disease. A) Positive correlation between fetal SVC flow and neonatal WM apparent diffusion coefficient. B) Negative correlation between fetal ascending aortic oxygen saturation and neonatal WM T2-score. C) Negative correlation between fetal oxygen consumption and neonatal WM T2-score. Pearson's r and p-values are in accompanying boxes.

Ascending aortic oxygen saturation (AAoSO_2_), VO_2_ and CVO_2_ were significantly lower in the CHD group (49% vs. 58%, p = 0.02; 5.03 ml/min/kg vs. 7.12 ml/min/kg, p= 0.02 and 2.98 ml/min/kg vs. 4.23 ml/min/kg, p=0.03, respectively). Although mean SVC flow (a surrogate for cerebral flow) was higher in CHD fetuses, the difference was not statistically significant. However, there was a moderately strong correlation between SVC flow and mean cerebral WM-ADC (Pearson's r =0.38, p= 0.012) (Figure 1a). ADC was higher in CHD neonates in the frontal WM (by 6.9 %, p= 0.008), parietal WM (by 5.9 %, p= 0.02), and CSO (by 2.3 %, not statistically significant). Visual T2-score was significantly higher in CHD neonates (3.08 vs. 1.16, p=0.002), with a negative correlation with AAoSO_2_ (r = -0.33, p= 0.04) and VO_2_ (r= -0.47, p= 0.005) (Figure 1b and 1c).

## Conclusions

We conclude that neonatal WM abnormalities in CHD are due to reduced fetal oxygenation. The correlation between more severe WM abnormality and elevated SVC flow, a known marker of acute fetal hypoxia (the "brain sparing" response) is further evidence that WM changes are driven by reduced cerebral oxygenation in utero.

## Funding

This study was funded through an operating grant from the CIHR and a PHN New Scholar Award (NIH).

**Table 1:** Table 1

		NORMAL (N= 25)	CHD (N= 15)	p-value
Age at MRI (Days)		5.24	4.93	0.85
Corrected gestational age at MRI (Weeks)		39.9	39	0.06
White matter T2-score (0 - 6)		1.16	3.08	0.002
ADC (x 10-6 mm2/sec)	Centrum semiovale	1461.7	1494.3	0.20
	Frontal deep white matter	1716.6	1818.2	0.02
	Parietal deep white matter	1668.1	1783.3	0.008
SVC flow (ml/min/kg)		134.4	144.6	0.43
AAoSO2 (%)		58	49	0.02
VO2 (ml/min/kg)		7.12	5.03	0.02
CVO2 (ml/min/kg)		4.23	2.98	0.03

AAoSO2 = Ascending aortic oxygen saturation; ADC = Apparent diffusion coefficient; CHD = Congenital heart disease; CVO2 = Cerebral oxygen consumption; SVC = Superior vena cava; VO2 = Fetal oxygen consumption.
